# Noise Improvement of a-Si Microbolometers by the Post-Metal Annealing Process

**DOI:** 10.3390/s21206722

**Published:** 2021-10-10

**Authors:** Jaesub Oh, Hyeong-sub Song, Jongcheol Park, Jong-Kwon Lee

**Affiliations:** 1 Division of Nano Convergence Technology, National NanoFab Center, Daejeon-si 34141, Korea; jsoh@nnfc.re.kr (J.O.); jcpark@nnfc.re.kr (J.P.); 2 Foundry Business, Samsung Electronics Co., Suwon-si 18448, Korea; hs2020.song@samsung.com; 3 Division of Energy and Optical Technology Convergence, Cheongju University, Cheongju-si 28503, Korea

**Keywords:** microbolometer, amorphous Si, noise characteristic, metal annealing, deutrium

## Abstract

To realize high-resolution thermal images with high quality, it is essential to improve the noise characteristics of the widely adopted uncooled microbolometers. In this work, we applied the post-metal annealing (PMA) process under the condition of deuterium forming gas, at 10 atm and 300 °C for 30 min, to reduce the noise level of amorphous-Si microbolometers. Here, the DC and temperature coefficient of resistance (TCR) measurements of the devices as well as *1/f* noise analysis were performed before and after the PMA treatment, while changing the width of the resistance layer of the microbolometers with 35 μm or 12 μm pixel. As a result, the microbolometers treated by the PMA process show the decrease in resistance by about 60% and the increase in TCR value up to 48.2% at 10 Hz, as compared to the reference device. Moreover, it is observed that the noise characteristics are improved in inverse proportion to the width of the resistance layer. This improvement is attributed to the cured poly-silicon grain boundary through the hydrogen passivation by heat and deuterium atoms applied during the PMA, which leads to the uniform current path inside the pixel.

## 1. Introduction

Infrared (IR) sensors detect electromagnetic waves in the range of 0.75 to 1000 μm, which are easy to pass through various media due to their long wavelength and have no sensitivity to the human eye. Here, thermal-type IR sensors use a change in the physical properties of a material according to temperature [1,2,3,4,5]. Since most of these thermal sensors operate at room temperature, they do not require a vacuum for cooling. In particular, microbolometers use the principle that the resistance of the material varies according to the temperature [6,7]. Since their process is relatively simple and can be manufactured with high yield and low cost [8,9,10], the microbolometers attracted much attention in the IR sensing field. Therefore, they have been used for various civilian and military applications, such as night vision system, security survey system, building diagnostic, biomedical thermography, etc. [10,11,12,13,14]. Recently, their applications are expanding throughout the industry as they are adopted in mobile products linked with applications that can capture IR images anytime and anywhere [10,15,16].

To achieve high performance of the microbolometers for thermo-sensing applications, their essential characteristics are low noise, large temperature coefficient of resistance (TCR), and low resistivity. In this regards, amorphous-Si (a-Si), vanadium oxide, and some metals have been adopted as a resistive layer of the microbolometer [1,7]. Particularly, the a-Si based microbolometers are preferential for commercializing the thermal imaging system [17,18], due to their high TCR and full compatibility with the Si-CMOS processing technology. However, the a-Si layer exhibits high resistivity and considerable thermal noise due to the operation at room temperature [19,20], which leads to the lower signal-to-noise ratio (S/N) than that of the cooling-type quantum IR sensors. It is noted that the noise characteristic of the a-Si based microbolometer is affected by doping, hydrogeneration, and structure of the a-Si resistive layer. For non-degenerated a-Si materials, their electrical noise was attributed to phonon assisted hopping [21]. In hydrogenated a-Si (a-Si:H) materials, the noise spectrum related to fluctuations in conductance normally follows a *1/f* power law [22]. It was reported that relative noise powers of 290 to 430 K in intrinsic a-Si:H films are associated with generation-recombination noise [23]. Moreover, for p-type a-Si:H films, the frequency exponent is about 1 at room temperature and increases to 1.4 at 400 K [24]. Furthermore, the *1/f* noise for boron doped p-type a-Si:H increased in proportion to hydrogen dilution [25]. Therefore, it is necessary to reduce the aforementioned noises of the a-Si based microbolometers to realize the high-resolution thermal image with high quality [26]. In this respect, P. Sharma et al. recently reported TCR values of 6–7%/K along with *1/f* noise constant of 10^−12^ in a porous Si-thin film with an optimized porosity of 60–75%, by passivating the surface at 600 °C to stabilize the film against atmospheric oxidation [27]. Moreover, there were reports on the results of comparing TCR values and *1/f* noise characteristics according to the crystal structure of boron-doped hydrogenated mixed-phase silicon films for the IR device application [28,29]. In particular, C. Shin et al. showed the TCR value of 1–3%, sheet resistance of 3–61.4 MΩ/sq, and *1/f* noise of 4 × 10^−10^/Hz for the crystalline volume fraction (7–17%) of films [29].

In this study, the post-metal annealing process was adopted to improve the thermal noise characteristics of a-Si microbolometers with 35 μm or 12 μm pixel. To quantify the variations in thermal noise depending on the resistance of the a-Si layer, we fabricated the microbolometers with different widths of the resistance layer by varying the pattern spacing of the absorbing TiN layer formed on the pixel. Then, by measuring the DC and TCR characteristics and analyzing the low-frequency noise before and after the PMA process, it is observed that the noise level of the microbolometers treated with the PMA process is noticeably reduced along with decreased resistance and increased TCR value, as compared to the reference devices. Thereafter, we described the effect of the PMA process on the improvement of the a-Si based microbolometers.

## 2. Experimental Section

### 2.1. Fabrication of a-Si Microbolometers

**Figure 1:**
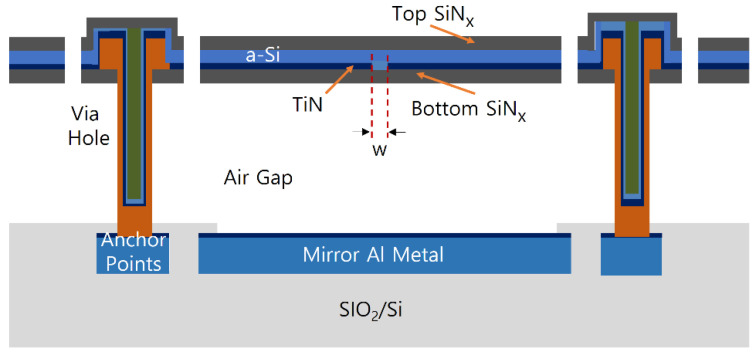
Schematic cross-sectional view of the a-Si microbolometer unit pixel. Here, SiN_x_ is silicon nitride deposited by the PECVD, and w denotes the pattern spacing of the TiN absorption layer.

**Figure 2:**
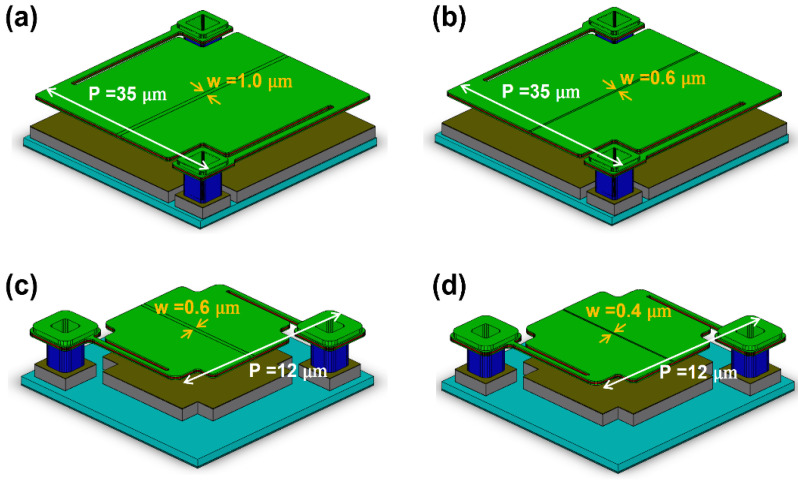
Schematic 3D views of the four different types of a-Si microbolometer unit pixels. (**a**) P35-S10, (**b**) P35-S06, (**c**) P12-S06, and (**d**) P12-S04.

Figure 1 shows the schematic of a-Si microbolometer unit pixel with an air gap of 2 μm. The IR reflective mirror is patterned on the Si substrate where the read-out integrated circuit (ROIC) for IR detection is formed, and the absorber consisting of a-Si layer and TiN layer sandwiched with the SiN_x_ layers is formed at a distance of λ/4 away from the top of the substrate. Here, the air-gap structure is applied between the bottom reflector and the absorption layer to maximize the absorption within the IR-sensitive layers through multiple reflection at an incident wavelength of λ, as well as thermally isolate the absorbing layers. The absorber is supported by the anchors formed on both sides, and a metal pad is formed between the anchor and the substrate. In the IR-sensitive layers, the temperature of the absorbing TiN layer increases through absorption of incident IR light, and the electrical resistance of the a-Si layer changes according to the temperature increase due to the heat transferred from the absorption layer. The anchor electrically connects the resistance layer and the ROIC circuit, and the ROIC detects the amount of absorbed IR radiation by measuring the resistance change according to the temperature. In this study, the microbolometers with different widths of the a-Si resistance layer were adopted by varying the pattern spacing (S-TiN) of absorbing TiN layer formed on the pixel, as schematically shown in Figure 2: The pixel size of 35 μm with TiN pattern spacing of 1.0 μm (P35-S10), pixel size of 35 μm with TiN pattern spacing of 0.6 μm (P35-S06), pixel size of 12 μm with TiN pattern spacing of 0.6 μm (P12-S06), and pixel size of 12 μm with TiN pattern spacing of 0.4 μm (P12-S04).

**Figure 3:**
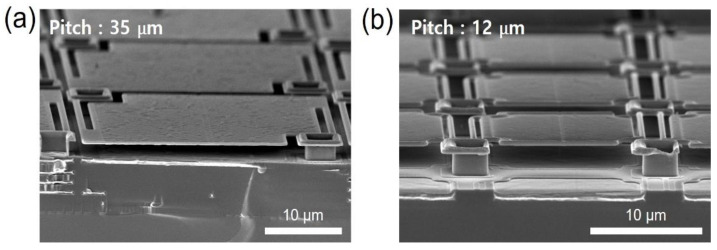
The FESEM tilted views of the fabricated a-Si microbolometers with a pixel pitch of (**a**) 35 and (**b**) 12 um, where the pattern spacing of the absorbing TiN layer was varied from 0.4 to 1 μm.

These resistive microbolometer detectors were fabricated using the surface micro-electro-mechanical system (MEMS) technology, which is compatible with a completely dry and fully CMOS process. Firstly, the reflection layer of Ti/Al/TiN with a thickness of 700 nm was deposited on a SiO_x_/Si substrate using sputtering equipment, and the bottom mirror and the lower electrode were patterned by etching. After planarizing the SiO_x_ layer deposited on the lower electrode, the sacrificial amorphous-carbon layer (ACL) with 2 μm thickness was deposited using the plasma-enhanced chemical vapor deposition (PECVD) on the metal pad (anchor point) and metal reflector of the substrate. The first passivation layer with 150 nm SiN_x_ was deposited on the sacrificial layer using the PECVD and patterned with the reactive ion etching (RIE) system to make via-holes with 4 μm diameter for 35 μm pixel or 2 μm diameter for 12 μm pixel up to the lower electrode. Then, the anchor metal layer of 300 nm TiN and the absorption layer of 15 nm TiN were deposited by the sputter system and selectively etched with the RIE systems. Here, the pattern spacing (S-TiN) of absorbing TiN layer formed in the pixel was varied from 0.4 to 1.0 μm. The boron-doped a-Si film was deposited as the resistance layer with 100 nm thickness, followed by deposition of 150 nm SiN_x_ as the second passivation layer. The structure layers were then patterned with the RIE system to form the shape with the thermally isolated legs. Finally, the microbolometer structure was suspended by removing the sacrificial ACL using an O_2_ plasma process. The field-emission scanning electron microscopy (FESEM) images for the fabricated a-Si microbolometers with a pixel pitch of 35 μm and 12 μm are shown in Figure 3a,b, respectively.

### 2.2. DC, TCR Measurements, and Noise Analysis

In order to observe the change in the performance of the a-Si microbolometer through the PMA process, we measured the electrical characteristics of the microbolometers before and after the PMA. All of the measurements were taken in a noise shielded room to block external interference. Firstly, DC, TCR, and noise characteristics of the microbolometers were measured to obtain reference data before the PMA process. The DC measurement was performed by applying 0 V to the cathode and sweeping 0 to 3 V to the anode. The TCR measurements were conducted under four different conditions at 25 °C intervals from 25 to 100 °C and extracted TCR values using the formula ***a*** = ∆ln***R***/∆***T*** [**1**/°C]. The 1/f noise characteristics were measured after applying 0 V to the cathode and 3 V to the anode. The PMA process was performed under the conditions of deuterium (D_2_) forming gas (N_2_ 96%, D_2_ 4%), at 10 atm and 300 °C for 30 min. To confirm the characteristic change of the microbolometers after the PMA process, the DC, TCR, and noise characteristics were measured under the same conditions as before applying the PMA process.

## 3. Results and Discussion

### 3.1. Electrical Characteristics of the a-Si Microbolometers before the PMA Process

**Figure 4:**
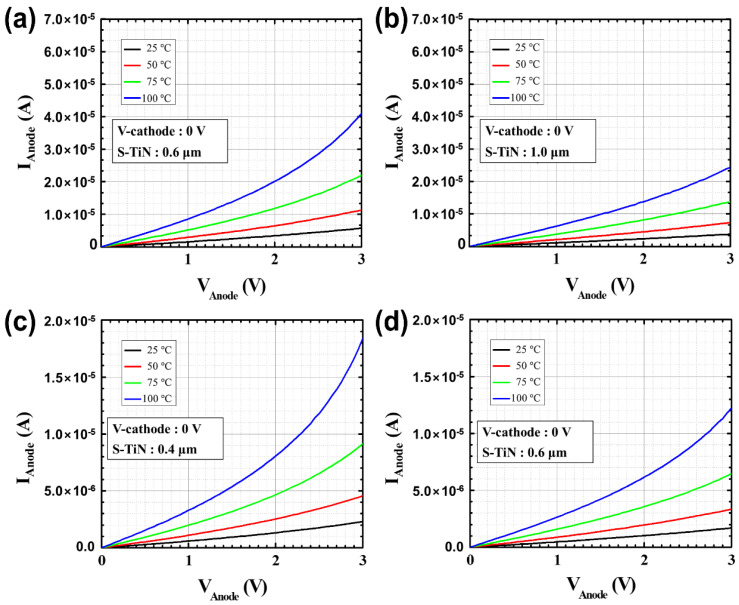
Measured DC characteristics according to the temperature (25, 50, 75, and 100 °C) of the four types of a-Si microbolometers: (**a**) P35-S06, (**b**) P35-S10, (**c**) P12-S04, and (**d**) P12-S06. Here, S-TiN stands for the pattern spacing of TiN layer.

**Figure 5:**
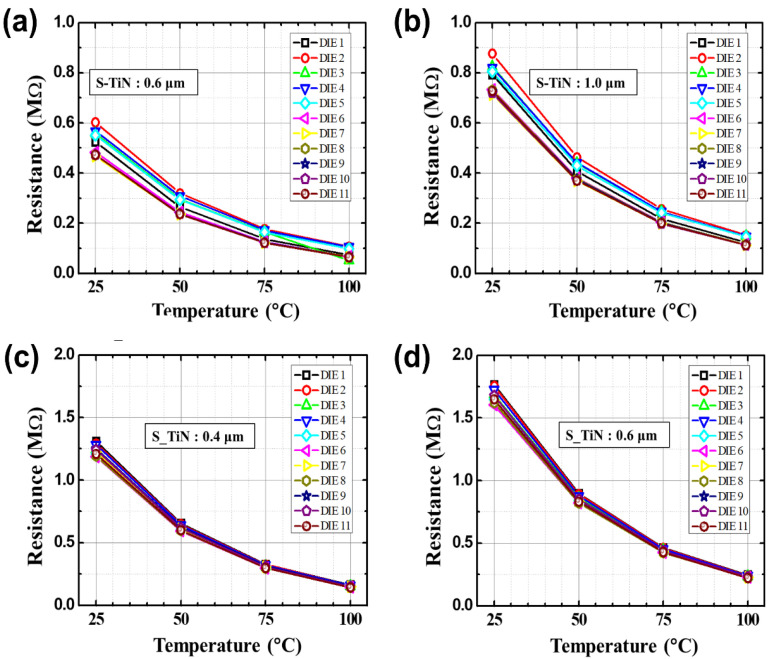
The resistance variations as a function of temperature for the four types of a-Si microbolometers: (**a**) P35-S06, (**b**) P35-S10, (**c**) P12-S04, and (**d**) P12-S06. Here, DIE 1 to DIE 11 represent the microbolometers with the same structure fabricated in different locations on the substrate, and the S-TiN stands for the pattern spacing of TiN layer.

**Figure 6:**
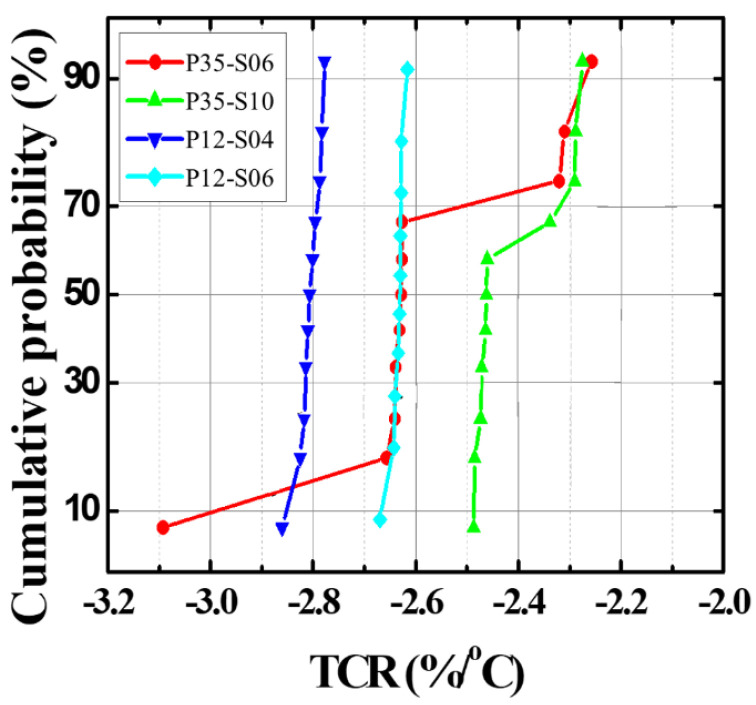
Cumulative probability curves of TCR extracted from the 11 devices for each of the four types of a-Si microbolometers.

Before applying the PMA process, the DC and low-frequency noise characteristics were measured depending on the temperature of the microbolometers for different widths of the resistance layer. Figure 4a,b shows the DC characteristics for each temperature according to the pattern spacing (S-TiN) of TiN layer of the a-Si microbolometers with a pixel size of 35 μm. It is confirmed that *I_anode_* is inversely proportional to the pattern spacing of TiN layer, corresponding to the pattern width of the resistance layer, while it is proportional to the temperature increment. These characteristics show the same trend when the pixel size of the microbolometer decreased to 12 μm, as shown in Figure 4c,d. Table 1 summarizes the anode current at 3 V for the *V_anode_* as temperature changes from 25 to 100 °C for the four types of microbolometers: P35-S06, P35-S10, P12-S04, and P12-S06. As aforementioned, *I_anode_* decreases as the pattern spacing of the TiN layer increases, while *I_aonde_* increases as the measurement temperature increases. In the case of the devices (P35-S06 and P12-S06) with the same pattern spacing of the TiN layer, the I_anode_ is not at the same current level, since each part such as the leg, anchor, and pixel size of the microbolometer is different. Additionally, the narrower the pattern spacing of TiN layer, the higher the noise level. Then, the resistance variations of the microbolometers as a function of temperature are presented in Figure 5, which was extracted from the I-V characteristics (Figure 4). The measured results for the 11 microbolometers of the four types (P35-S06, P35-S10, P12-S04, P12-S06) reveal that the resistance of the microbolometers decreases as the measurement temperature increases. A graph showing the cumulative curve of TCR extracted from the 11 microbolometers for each split condition is shown in Figure 6. The microbolometers with a pixel pitch of 12 μm exhibit relatively larger TCR values above the cumulative probability of 20% than the cases with the 35 μm pixel. In particular, the microbolometers with a pixel pitch of 12 μm show a relatively constant TCR change, while the ones with a pixel pitch of 35 μm exhibit a slightly large variation in the TCR values. It is noted that the total resistance of the microbolometer is determined by the resistance (*R_s_*) of the a-Si layer in the pixel as well as the resistance (*R_end_*) of the legs including the via-holes. The contribution of *R_s_* and *R_end_* to total resistance varies depending on the pixel structure, and the change in the *R_s_* depending on temperature mainly contributes the TCR value of total resistance. In our fabricated microbolometers, the contribution of the *R_s_* to the total resistance in the 12 μm pixel is reduced compared to the cases of 35 μm pixel, while the contribution of the *R_end_* to the total resistance increases. Table 2 compares the values of the midpoint (50%) of the cumulative curve shown in Figure 6. It is observed that the TCR value increases as the pattern width of the resistance layer decreases. The 12-μm pixel microbolometers (P12-S04) with TiN pattern spacing of 0.4 μm show TCR of 0.18%/°C more than the ones (P12-S06) with 0.6 μm, and the 35-μm pixel microbolometers (P35-S06) with TiN pattern spacing of 0.6 μm exhibit TCR of 0.17%/°C more than the ones (P35-S10) with 1.0 μm. Therefore, it is seen that the device with a smaller pattern spacing of TiN layer has lower resistance, resulting in higher TCR.

**Figure 7:**
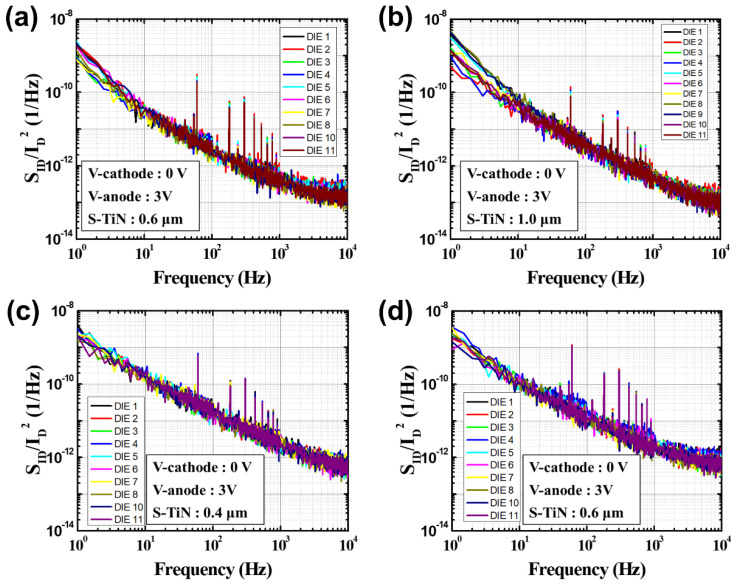
Noise characteristics normalized with *I_anodes_* of the four types of a-Si microbolometers: (**a**) P35-S06, (**b**) P35-S10, (**c**) P12-S04, and (**d**) P12-S06.

**Figure 8:**
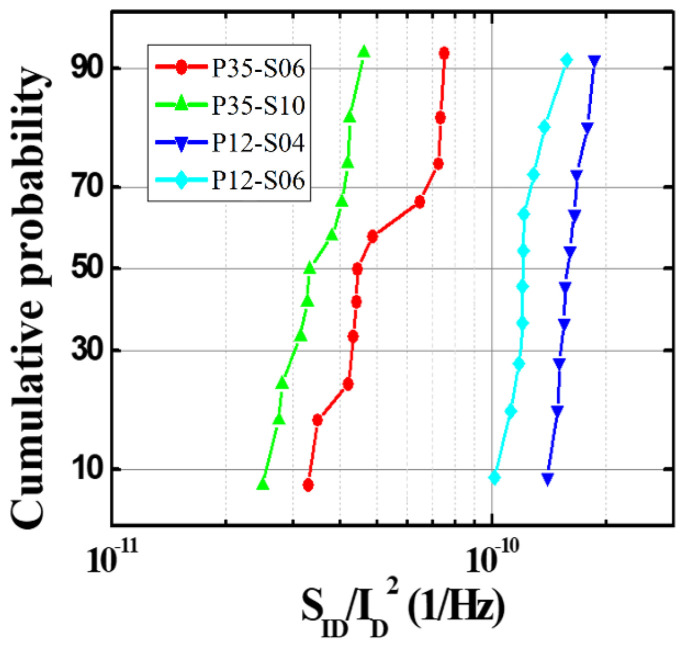
Cumulative probability curves of the low-frequency noise characteristics extracted at 10 Hz from the 11 devices for each of the four types of a-Si microbolometers.

The noise characteristics of the devices with the pixel pitch of 35 μm or 12 μm depending on the pattern spacing (S-TiN) of the TiN layer were measured by the *I_ano__de_* at 3 V, as shown in Figure 7. Here, *S_ID_* indicates the noise characteristic, and its unit is [A^2^/Hz]. It is noted that the noise characteristic (*S_ID_*) was normalized with the anode current ($I_{D}^{2}$) to compare the performance before and after the PMA treatment, since the *I_anode_* of the microbolometers treated by the PMA process increases. Therefore, the unit of *S_ID_*/$I_{D}^{2}$ in Figure 7 is [1/Hz]. The noise characteristics obtained from the four types of microbolometers show *1/f* characteristics in all of the cases. Figure 8 shows the cumulative curves of the low-frequency noises extracted at 10 Hz, which is the standard for low-frequency noise characteristics. It shows that the magnitude of the low-frequency noise at 10 Hz tends to decrease as the pattern spacing of TiN layer increases. Table 3 presents the magnitudes of low-frequency noise at the midpoint (50%) of the cumulative curves for the four types of microbolometers. The microbolometers with a pixel size of 12 μm have a higher noise level than those with a pixel size of 35 μm. In the case of a 35 μm-pixel microbolometer with TiN pattern spacing of 0.6 μm, it is observed that the noise level is approximately 25.2% larger than that with TiN spacing of 1 μm. Additionally, the same phenomenon occurs in the microbolometer with a smaller pixel size of 12 μm. In the case of the microbolometer with TiN size of 0.4 μm, it is observed that the noise level of about 22.5% is greater than that of the microbolometer with TiN spacing of 0.6 μm. Therefore, the smaller the pixel size and the narrower the pattern spacing of TiN layer, the higher the noise level.

### 3.2. Electrical Characteristics of the a-Si Microbolometers after the PMA Process

**Figure 9:**
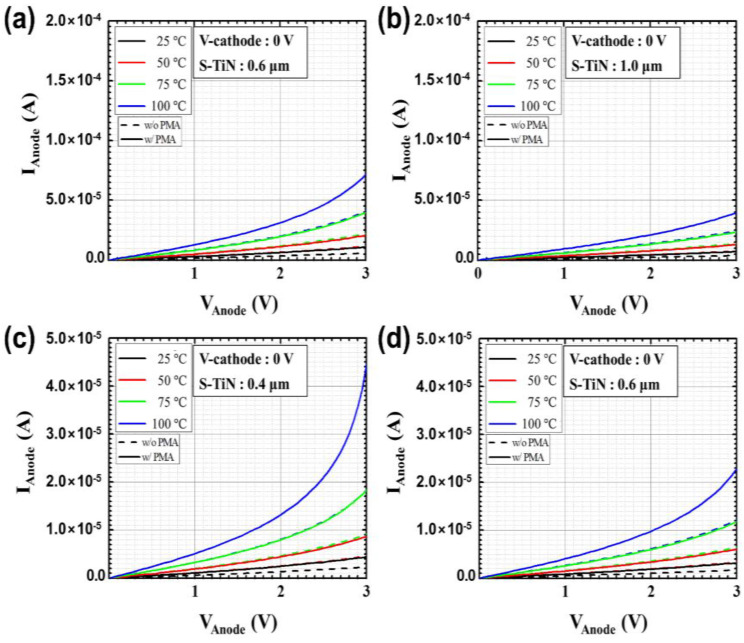
Measured DC characteristics according to the temperature (25, 50, 75, and 100 °C) of the four types of a-Si microbolometers before (dotted lines) and after (solid lines) applying the PMA process: (**a**) P35-S06, (**b**) P35-S10, (**c**) P12-S04, and (**d**) P12-S06. Here S-TiN stands for the pattern spacing of TiN layer.

**Figure 10:**
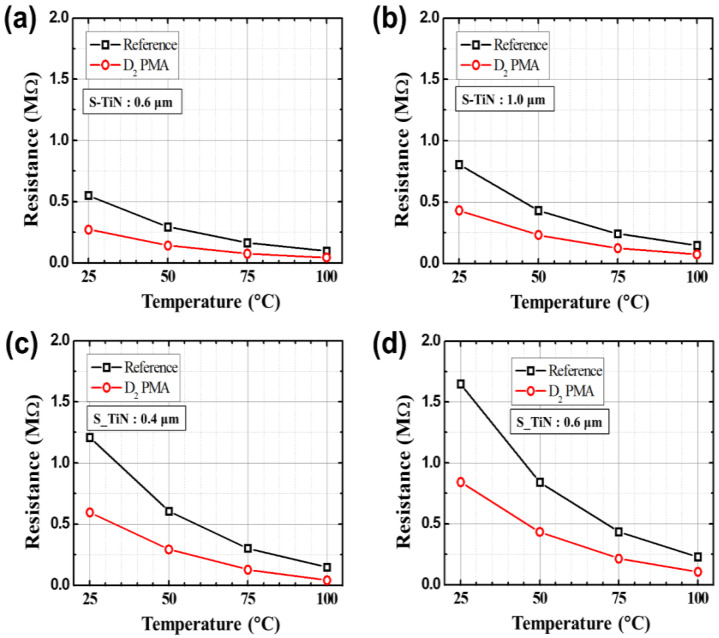
The resistance variations as a function of temperature for the four types of a-Si microbolometers before and after applying the PMA process: (**a**) P35-S06, (**b**) P35-S10, (**c**) P12-S04, and (**d**) P12-S06. Here, the S-TiN stands for the pattern spacing of TiN layer.

**Figure 11:**
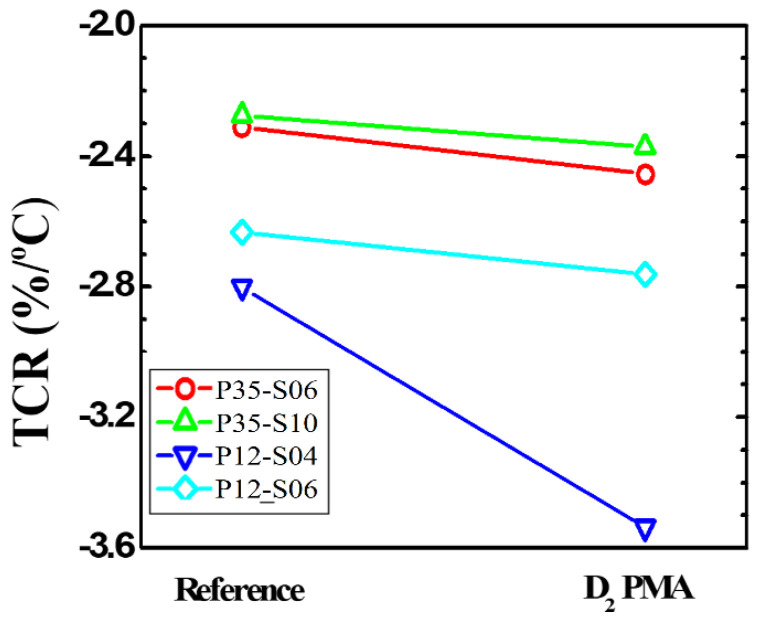
Comparison of TCR values of the four types of a-Si microbolometers treated with the PMA process and the reference devices.

The DC and TCR measurements as well as the noise analysis were performed for the a-Si based microbolometers treated with the PMA process under the condition of D_2_ forming gas, at 10 atm and 300 °C for 30 min. The DC characteristics of the microbolometers measured after the PMA process are shown in Figure 9, where the measured anode currents (*I_anode_*) before applying the PMA process were also included as reference data. By performing the PMA treatment, it is observed that the *I_anode_* shows a tendency to increase compared to the reference in all of the temperature conditions, and the *I_anode_* shows an average increase of about 60% compared to the reference at 3 V. Figure 10 shows the change in the resistance value according to the temperature change of the four types of microbolometers subjected to the PMA in the D_2_ condition. It is clearly observed that the resistance decreases by performing the PMA process to the microbolometers. In particular, the significant decrease is seen in the case of the device with a pixel pitch of 12 μm, as shown in Figure 10c,d. The TCR values extracted from the measured data are shown in Figure 11 and numerically summarized in Table 4. The TCR values of all the microbolometers increase as compared to the reference devices, and the microbolometers with a smaller pattern spacing of TiN layer exhibit higher increase in TCRs than those with a larger one. In particular, it shows a significant increase by 26.3% for the 12 μm-pixel microbolometers with TiN pattern spacing of 0.4 μm.

**Figure 12:**
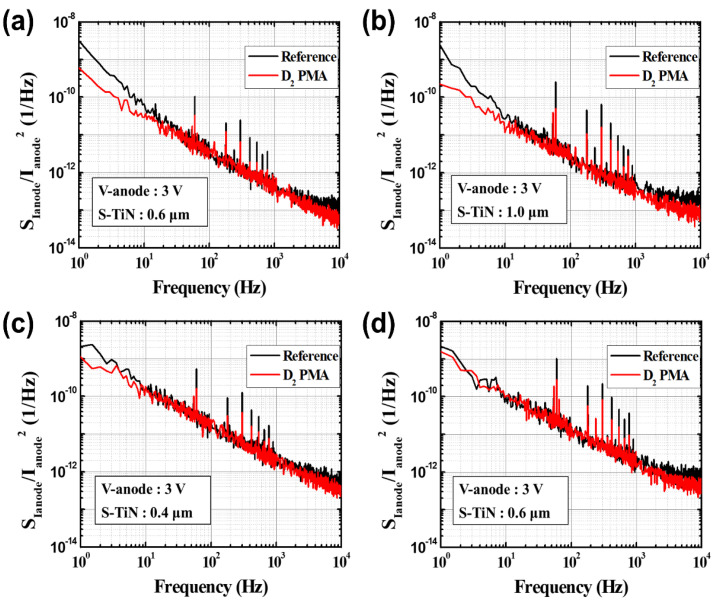
Comparison of noise characteristics normalized with I_anodes_ of the four types of a-Si microbolometers before (black solid lines) and after (red solid lines) applying the PMA process: (**a**) P35-S06, (**b**) P35-S10, (**c**) P12-S04, and (**d**) P12-S06.

**Figure 13:**
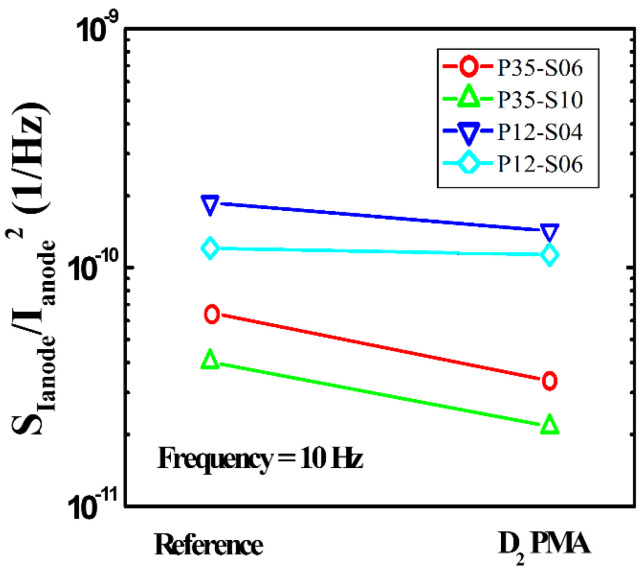
Comparison of low-frequency noise characteristics at 10 Hz of the a-Si microbolometers treated with the PMA process and the reference devices.

The noise characteristics of the microbolometer subjected to the PMA process under the D_2_ condition are shown in Figure 12. By normalizing the noise data with respect to the anode current, it is observed that the noise levels of all the microbolometers treated by the D_2_ PMA process are lowered in the frequency range compared to those of the reference devices. Figure 13 shows the variation in low-frequency noise characteristics at 10 Hz of the a-Si microbolometers before and after the PMA process. Table 5 presents the noise levels at 10 Hz of the four types of devices applied with the PMA process and the increase/decrease rate with respect to the reference devices. The microbolometers with a 35 μm pixel exhibit more improved noise levels than those with a 12 μm pixel. In addition, it reveals that the smaller the pattern spacing of the TiN layer, the higher the noise reduction rate compared to the reference. In particular, the microbolometers with 35 μm pixel size show a significant decreased rate of 48.2%, and 46.4% for the TiN pattern spacing of 0.4 and 1.0 μm, respectively, in terms of noise levels at 10 Hz with respect to the reference devices. Meanwhile, the microbolometers with 12 μm pixel pitch show a decreased rate of 23.7 and 6.34% for the TiN pattern spacing of 0.4 and 0.6 μm, respectively.

As a result of the measured data, by performing the PMA process under the condition of D_2_ forming gas, at 10 atm and 300 °C for 30 min, it is observed that the resistance decreased by about 60% compared to the reference microbolometer, and the TCR values increased up to 48.2%. Therefore, the fabricated a-Si microbolometers treated by the PMA process show TCR values of 2.4~3.5%/K and *1/f* noise levels of around 10^−11^/Hz, which are comparable to those of the previously reported literatures [27,28,29]. Moreover, it is confirmed that the noise characteristics are improved in inverse proportion to the patten width of resistance layer. This noise improvement can be attributed to the cured poly-silicon grain boundary through hydrogen passivation by heat and D_2_ atoms applied during the PMA process [30,31]. Furthermore, according to the previous report [25], the *1/f* noise for boron doped p-type a-Si increases in proportion to hydrogen dilution. Therefore, the hydrogen passivation by heat and deuterium during the PMA process contributes to the decrease in the *1/f* noise. Consequently, the PMA process applied to the a-Si microbolometers makes the current path uniform inside the pixels.

## 4. Conclusions

We demonstrated the noise improvement of the a-Si microbolometer by utilizing the PMA process under D_2_ forming gas, at 10 atm and 300 °C for 30 min. A study on the variation in thermal noise characteristics according to the change in the pattern width of the a-Si resistance layer were conducted for the four types of microbolometers with the pixel size of 35 μm or 25 μm. The measured DC, TCR, and low-frequency characteristics of the devices treated by the PMA process reveal the reduced resistance of ~60% along with the increased TCR value up to 48.2%, in comparison with those of the reference devices. Here, the cured Si grain boundary through hydrogen passivation by heat and deuterium atoms during the PMA process makes the current path uniform inside the pixel. Moreover, the measured results show that the noise reduction rate increases as the pattern width of resistance layer decreases. Therefore, the proposed PMA process is expected to be the simple and cost-effective way to realize the high-resolution a-Si microbolometer with significantly reduced noise levels.

## Figures and Tables

**Table 1:** The anode current at 3 V according to the temperature of the four types of a-Si microbolometers.

Temperature	P35-S06	P35-S10	P12-S04	P12-S06
25 °C	5.72 × 10^−6^ A	3.79 × 10^−6^ A	2.29 × 10^−6^ A	1.70 × 10^−6^ A
50 °C	1.13 × 10^−5^ A	7.36 × 10^−6^ A	4.57 × 10^−6^ A	3.35 × 10^−6^ A
75 °C	2.20 × 10^−5^ A	1.38 × 10^−5^ A	9.14 × 10^−6^ A	6.46 × 10^−6^ A
100 °C	4.09 × 10^−5^ A	2.44 × 10^−5^ A	1.84 × 10^−5^ A	1.22 × 10^−5^ A

**Table 2:** Comparison of values at the midpoint (50%) of the cumulative curves plotted in Figure 6 of the four types of a-Si microbolometers.

	P35-S06	P35-S10	P12-S04	P12-S06
TCR (%/°C)	−2.63	−2.46	−2.81	−2.63

**Table 3:** Comparison of values at the midpoint (50%) of the cumulative curves plotted in Figure 8 of the four types of a-Si microbolometers.

	P35-S06	P35-S10	P12-S04	P12-S06
S_Iaonde_/I_anode_^2^ (1/Hz)	4.44 × 10^−11^	3.32 × 10^−11^	1.56 × 10^−10^	1.21 × 10^−10^

**Table 4:** TCR values of the four types of a-Si microbolometers applied with the PMA process and the increase/decrease rate compared to the reference.

	P35-S06	P35-S10	P12-S04	P12-S06
TCR (%/°C)	−2.46	−2.37	−3.54	−2.76
Increase rates w.r.t reference	6.28%	4.18%	26.3%	4.94%

**Table 5:** Low-frequency noise characteristics of the four types of a-Si microbolometers applied with the PMA process extracted at 10 Hz and the increase/decrease rate compared to the reference.

	P35-S06	P35-S10	P12-S04	P12-S06
Noise level at 10 Hz (1/Hz)	3.36 × 10^−11^	2.16 × 10^−11^	1.42 × 10^−10^	1.13 × 10^−10^
Decrease rates w.r.t reference	−48.2%	−46.4%	−23.7%	−6.34%

## Data Availability

Data and materials can be made available upon request to the authors.
